# GTDB-Tk: a toolkit to classify genomes with the Genome Taxonomy Database

**DOI:** 10.1093/bioinformatics/btz848

**Published:** 2019-11-15

**Authors:** Pierre-Alain Chaumeil, Aaron J Mussig, Philip Hugenholtz, Donovan H Parks

**Affiliations:** Australian Centre for Ecogenomics, School of Chemistry and Molecular Biosciences, The University of Queensland, St Lucia, QLD 4072, Australia

## Abstract

**Summary:**

The Genome Taxonomy Database Toolkit (GTDB-Tk) provides objective taxonomic assignments for bacterial and archaeal genomes based on the GTDB. GTDB-Tk is computationally efficient and able to classify thousands of draft genomes in parallel. Here we demonstrate the accuracy of the GTDB-Tk taxonomic assignments by evaluating its performance on a phylogenetically diverse set of 10 156 bacterial and archaeal metagenome-assembled genomes.

**Availability and implementation:**

GTDB-Tk is implemented in Python and licenced under the GNU General Public Licence v3.0. Source code and documentation are available at: https://github.com/ecogenomics/gtdbtk.

**Supplementary information:**

Supplementary data are available at *Bioinformatics* online.

## 1 Introduction

It has recently become possible to obtain thousands of draft bacterial and archaeal genomes directly from environmental- and human-associated samples (Anantharaman *et al.*, 2016; Parks *et al.*, 2017; Pasolli *et al.*, 2019). Accurate taxonomic classification of such genomes is a primary requirement for their analysis and essential for facilitating communication within the research community (Godfray, 2002). This is generally accomplished by manually inspecting the placement of genomes in 16S rRNA or concatenated protein phylogenies along with 16S rRNA percent identity or average nucleotide identity (ANI; Konstantinidis and Tiedje, 2005) statistics to support assignments to specific taxonomic ranks. This is a labour intensive and subjective endeavour (Coil *et al.*, 2019), and there is a lack of dedicated tools for classifying genomes with the notable exceptions of PhyloPhlAn (Segata *et al.*, 2013) and MiGA (Rodriguez-R *et al.*, 2018), which are currently based on the NCBI taxonomy (Federhen, 2015). Here we introduce the Genome Taxonomy Database Toolkit (GTDB-Tk), a computationally efficient toolkit that provides automated and objective taxonomic classification of bacterial and archaeal genomes by placing them into domain-specific, concatenated protein reference trees. GTDB-Tk determines taxonomic classifications consistent with the recently proposed rank-normalized GTDB taxonomy by using the same criteria of relative evolutionary divergence (RED) and ANI for establishing taxonomic ranks (Parks *et al.*, 2018, 2019).

## 2 Materials and Methods

### 2.1 Reference trees and taxonomies

GTDB-Tk uses the bacterial and archaeal reference trees, multiple sequence alignments, and taxonomy provided through the GTDB website (gtdb.ecogenomic.org). GTDB is updated bi-annually to incorporate the latest genomes in the NCBI Assembly database (Kitts *et al.*, 2016) and GTDB-Tk follows this update cycle. Results reported here are based on GTDB-Tk v0.3.2 and GTDB R04-RS89 where the reference trees span 23 458 bacterial and 1248 archaeal species.

### 2.2 Placement of genomes in reference trees

GTDB-Tk accepts genome assemblies as FASTA files, calls genes using Prodigal (Hyatt *et al.*, 2010), and identifies a set of 120 bacterial and 122 archaeal marker genes using HMMER (Eddy, 2011) as previously described (Parks *et al.*, 2018). Genomes are assigned to the domain with the highest proportion of identified marker genes. The selected domain-specific markers are aligned with HMMER, concatenated into a single multiple sequence alignment, and trimmed with the ∼5000 column bacterial or archaeal mask used by GTDB. Genomes are then placed into the domain-specific reference trees using pplacer (Matsen *et al.*, 2010).

### 2.3 Taxonomic classification

**Fig. 1.**
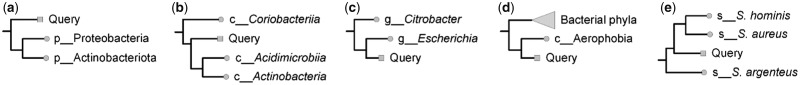
Illustrative examples of GTDB-Tk taxonomic assignments. (**a**) The position of the query genome in the reference tree alone may be sufficient to dictate its taxonomic assignment as in this example where it is necessarily a novel phylum. (**b**) Query genome represents a novel class within the phylum Actinobacteria. (**c**) Query genome will be classified as either a novel, basal *Escherichia* species or a novel genus in the family Enterobacteriaceae depending on its RED value. (**d**) Aerophobia is the only class within the Aerophobetota phylum and as such the query genome may be classified as the most basal order in Aerophobia, a novel class within the Aerophobetota, or a novel phylum depending on its RED value. (**e**) ANI is calculated between the query genome and the representative genomes for all *Staphylococcus* species. The query genome is assigned to the closest *Staphylococcus* species if the ANI is above the species ANI circumscription radius or is otherwise classified as a novel species.

Classification of a query genome is based on a combination of its placement in the GTDB reference tree, its RED (Parks *et al.*, 2018), and its ANI to reference genomes (Fig. 1 and Supplementary Fig. S1). In many cases the classification of a query genome is apparent from the topology of the tree (Fig. 1a and b). RED is used to resolve instances where rank assignments are ambiguous (Fig. 1c and d). Species assignments are established using ANI as calculated with FastANI (Jain *et al.*, 2017). Specifically, a query genome placed within a genus is assigned to the species of the closest reference genome with an alignment fraction >65% if it is within this species ANI circumscription radius (typically, 95%) as defined by the GTDB (Fig. 1e;Parks *et al.*, 2019). Otherwise, the query genome is classified as a novel species within the genus.

### 2.4 Requirements

GTDB-Tk is intended to run on a server with multiple CPUs and ≥128 GB of RAM. It can classify ∼1000 genomes per hour when using 64 CPUs. We recommend GTDB-Tk be applied to genomes estimated to be ≥50% complete with ≤10% contamination consistent with community standards for medium or higher quality single-amplified and metagenome-assembled genomes (MAGs; Bowers *et al.*, 2017).

## 3 Results

Here we evaluate the accuracy of GTDB-Tk classifications by applying it to a set of 10 156 phylogenetically diverse bacterial (9386 genomes) and archaeal (770 genomes) MAGs of varying genomic quality that were manually curated in GTDB R04-RS89. These genomes comprise the uncultivated bacteria and archaea (UBA; Parks *et al.*, 2017) dataset and its extension released as part of the GTDB. GTDB-Tk classification performance was evaluated using reference trees inferred *de novo* without the UBA genomes, resulting in MAGs that represent novel taxa at all taxonomic ranks (Table 1). Classifications for these MAGs took 14 hours wall time using 32 2.30 GHz Intel Xeon E5-2650 CPUs and required 100 GB of RAM. Of the 10 156 MAGs, 1071 (10.6%) did not have identical GTDB-Tk and GTDB classifications (Table 1 and Supplementary Tables S1–S3). However, only 8 (0.08%) MAGs were placed in the reference tree in a position resulting in a conflict between the GTDB and GTDB-Tk assignments (e.g. family Cycloclasticaceae versus Methylomonadaceae; Supplementary Fig. S2). GTDB-Tk predicted at least one more rank than expected for 644 (6.34%) MAGs (over-classified; e.g. o__ Methylococcales; f__Methylomonadaceae although the MAG belongs to a novel family in the GTDB; Supplementary Figs S2 and S3) and at least one fewer rank than expected for 419 (4.13%) MAGs (under-classified; e.g. c__Anaerolineae; o__[novel order] although the MAG belongs to o__Anaerolineales, an order present in the *de novo* GTDB reference tree; Supplementary Fig. S4). The UBA genomes are estimated to be ≥50% complete with ≤10% contamination and no systematic bias was observed in over- and under-classifications as a function of genome quality (Supplementary Fig. S5). As the placement of genomes in a reference tree is non-deterministic, we examined the reproducibility of taxonomic assignments on random subsets of 100 UBA genomes. None of the UBA genomes had a different taxonomic assignment across 50 independent trials (Supplementary Table S4).

**Table 1.** Classification performance on the 10 156 UBA genome dataset indicating the lowest rank for which classifications consistent with GTDB assignments can be obtained by GTDB-Tk

Rank	No. UBA genomes	Identical	Conflicting	Over-classified^a^	Under-classified^b^
Domain	20	15.0%	0%	85.0%	0%
Phylum	56	69.6%	0%	28.6%	1.79%
Class	130	56.2%	0%	40.8%	3.08%
Order	442	59.1%	0.45%	32.4%	8.14%
Family	1788	71.8%	0.22%	21.6%	6.42%
Genus	3927	92.6%	0.05%	0.74%	6.60%
Species	3793	99.9%	0%	0%	0.11%
Total	10 156	89.5%	0.08%	6.34%	4.13%

a GTDB-Tk provides more resolved classifications than GTDB (Supplementary Figs S2 and S3).

b GTDB-Tk provides less resolved classification than GTDB (Supplementary Fig. S4).

Inferred evolutionary relationships are impacted by the set of taxa being considered (Nabhan and Sarkar, 2012). This naturally leads to some degree of over- and under-classification when considering the UBA genomes individually as per GTDB-Tk instead of as a complete set of genomes as per the GTDB classifications (Supplementary Fig. S6). Over- and under-classifications also occur as a result of differences between the strict quantitative rules applied by GTDB-Tk and the taxonomic opinion of GTDB curators which use a relatively wide RED range to guide classifications (Parks *et al.*, 2018). For example, GTDB curators may elect to define two classes even when combining them into a single class would result in a RED value closer to the median RED for class level taxa (Supplementary Fig. S7A). This occurs for a number of reasons including favouring assignment of taxa to nodes with high support values and prioritizing the retention of established taxon names. The converse situation also occurs where manual curation may define a single taxon resulting in GTDB-Tk classifications being under-classified relative to the GTDB (Supplementary Fig. S7B).

Disagreement between GTDB-Tk and manual curation is most pronounced when considering genomes that represent either a basal class within an existing phylum or a novel phylum (Table 1). The topological relationships between deep lineages are often unsupported by bootstrap resampling and this necessitates that these lineages be defined as new phyla even when their RED values are more commensurate with being defined as a basal class (Supplementary Fig. S3). GTDB-Tk only takes into account tree topology for user genomes as bootstrap resampling is computationally prohibitive and consequently may over-classify genomes relative to manual curation based on unsupported affiliations of user genomes to reference taxa.

The overall result of the benchmarking is that GTDB-Tk classifications are largely consistent with manual curation (89.5%), especially at lower ranks. Importantly, the great majority of disagreements are confined to a single rank difference (1057 of 1071 cases; 98.7%). Nonetheless, studies primarily concerned with elucidating evolutionary relationships or supporting taxonomic reclassifications should use GTDB-Tk as a guide to performing additional analyses constituting best practice in the field.

## 4 Summary

GTDB-Tk serves as a convenient means for the research community to classify the increasing numbers of microbial genomes recovered from metagenomic datasets. It has already been independently and positively evaluated for classification of MAGs (Coil *et al.*, 2019) and is available as an on-line resource through KBase (Arkin *et al.*, 2018) in addition to being a standalone tool. GTDB-Tk will serve as the basis for classifying new genomes incorporated into future GTDB releases, with curators adopting alternative classifications only when it accommodates known instabilities in inferred reference trees or to adhere to the taxonomic opinion of research groups making proposals that, while not in agreement with GTDB-Tk, satisfy the broader classification criteria underlying the GTDB.

## Supplementary Material

btz848_Supplementary_Data
