# Supplementary material for: GTDB-Tk: a toolkit to classify genomes with the Genome Taxonomy Database
Source: Bioinformatics. 2019 Nov 15;36(6):1925–7. doi: 10.1093/bioinformatics/btz848 (PMC7703759; doi:10.1093/bioinformatics/btz848)
Supplement: btz848_Supplementary_Data [file btz848_supplementary_data.zip › btz848-Suppl_Data/Chaumeil_GTDB_Tk_supp_material.revised.docx]

**Supplemental Information**

**GTDB-Tk: A toolkit to classify genomes with the Genome Taxonomy Database**

Pierre-Alain Chaumeil, Aaron J. Mussig, Philip Hugenholtz, Donovan H. Parks

**Supplemental Figures**


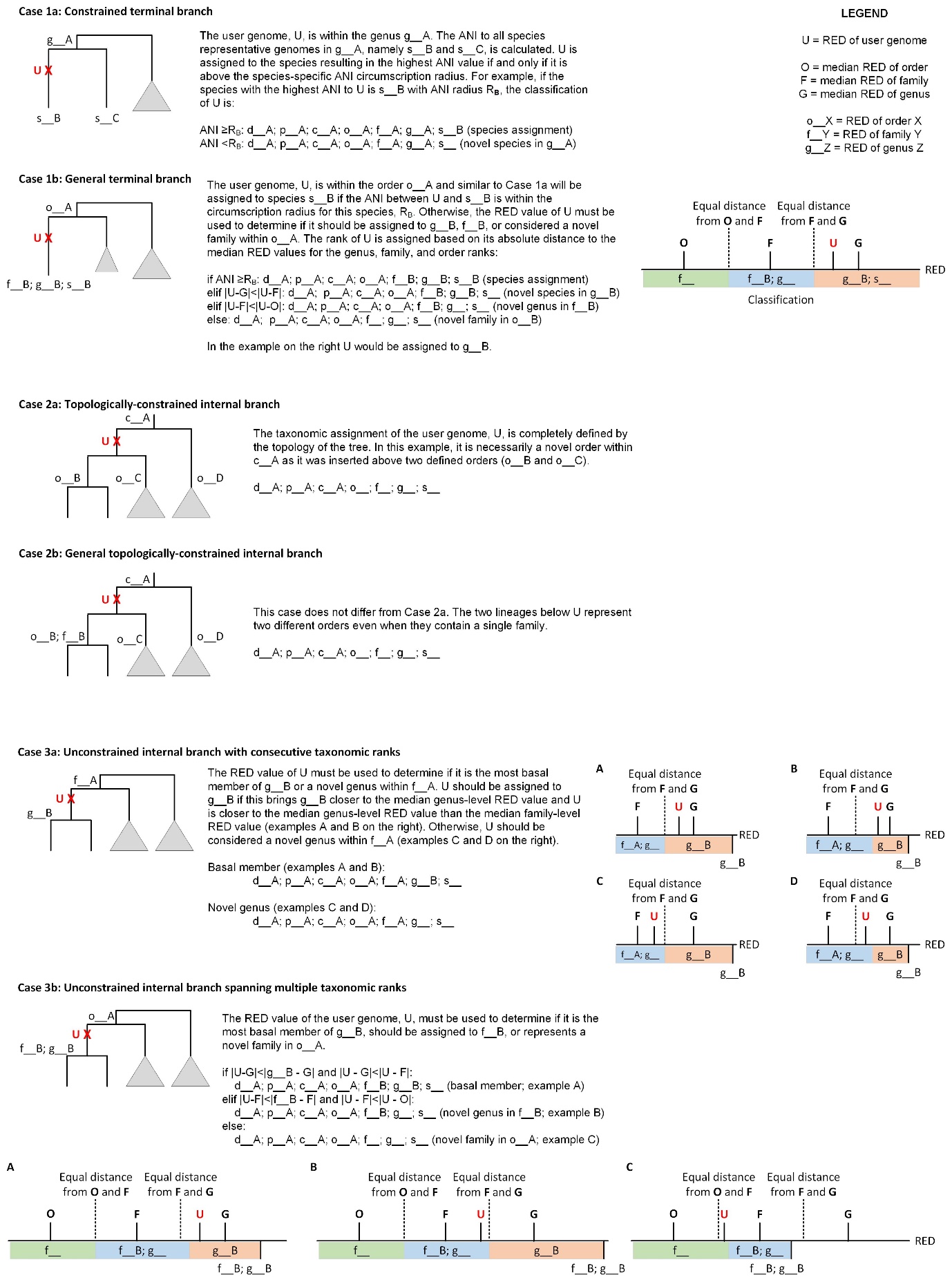


**Supplemental Figure S1**. Classification rules used by the GTDB-Tk. There are three possible cases that may occur when establishing the classification of a query genome. Each case is divided into two scenarios in order to provide a simple example of the rule followed by the general case that must be handled by GTDB-Tk.


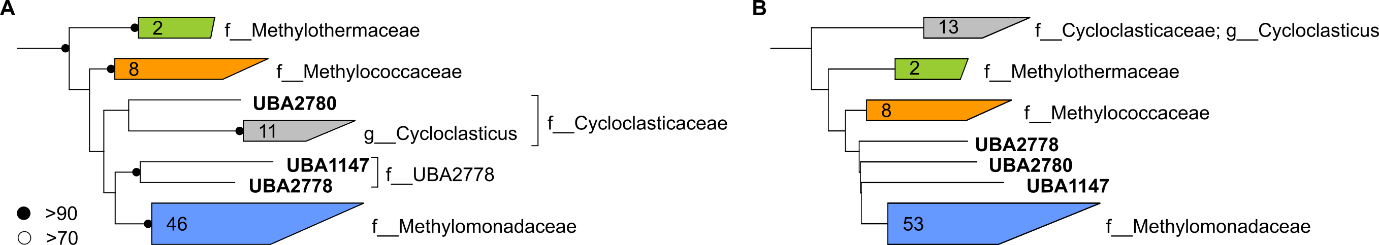


**Supplemental Figure S2**. Example of conflicting and overclassified GTDB-Tk assignments relative to GTDB classifications. The GTDB R04-RS89 reference (**A**) and GTDB-Tk classification (**B**) trees for families within the order *Methylococcales*. UBA2780 is a conflicting family assignment as it was classified to the family *Cycloclasticaceae* by GTDB curators and to the family *Methylomonadaceae* by GTDB-Tk. This conflicting assignment is a result of differences in tree topology and the resulting placement of the UBA2780 genome in the GTDB-Tk reference tree. UBA1147 and UBA2778 were overclassified order assignments as they were classified to the family f__UBA2778 by GTDB curators and to the family *Methylomonadaceae* by GTDB-Tk. This is overclassified at the rank of order as the taxon f__UBA2778 does not exist in the GTDB-Tk reference tree since it is comprised exclusively of UBA genomes. Consequently, the most specific GTDB-Tk classification in agreement with GTDB is to the order *Methylococcales*, and UBA1147 and UBA2778 should be considered as being from a novel family in order to agree with the GTDB classification.


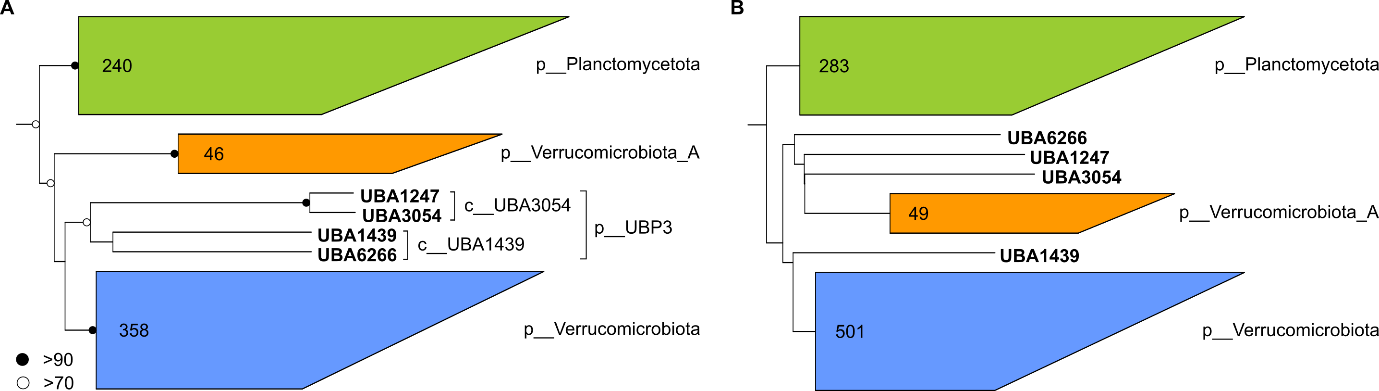


**Supplemental Figure S3**. Example of overclassified domain-level GTDB-Tk assignments relative to GTDB classifications. The GTDB R04-RS89 reference (**A**) and GTDB-Tk classification (**B**) trees for the phyla Planctomycetota, UBP3, Verrucomicrobiota, Verrucomicrobiota_A. UBA1247, UBA3054, UBA1439, and UBA6266 were assigned to the phylum UBP3 by GTDB curators. This phylum is comprised exclusively of UBA genomes and consequently this taxon is not defined within the GTDB-Tk tree. The 4 UBA genomes should be classified as belonging to a novel phylum in order to be in agreement with the GTDB classification. However, GTDB-Tk classified these genomes as basal members of the phyla Verrucomicrobiota_A and Verrucomicrobiota and thus are considered overclassification relative to the GTDB. These 4 UBA genomes were assigned to a novel phylum by GTDB curators due to the poor bootstrap support between the UBP3 and Verrucomicrobiota lineages. The poor support for the placement of the UBP3 lineage is reflected in the GTDB-Tk classification tree where 3 of the genomes associate more closely with Verrucomicrobiota_A than Verrucomicrobiota.


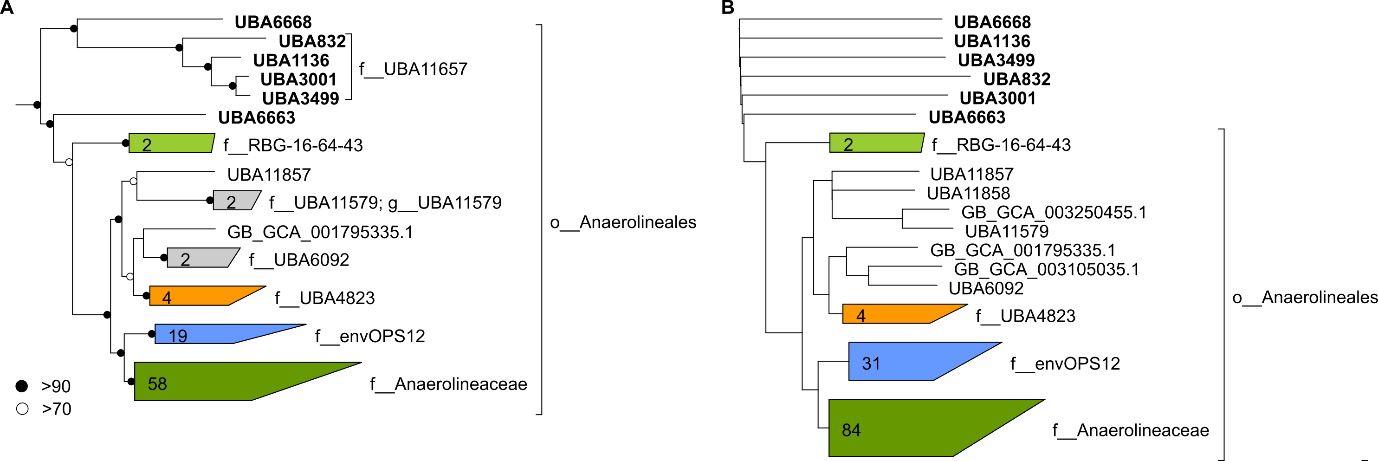


**Supplemental Figure S4**. Example of underclassified order-level GTDB-Tk assignments relative to GTDB classifications. The GTDB R04-RS89 reference (**A**) and GTDB-Tk classification (**B**) trees for the order *Anaerolineales*. UBA6668, UBA832, UBA1136, UBA3001, UBA3499, and UBA6663 were classified as basal families within the order *Anaerolineales* by GTDB curators. GTDB-Tk classified these genomes as novel orders within the class *Anaerolineae*. Since the order *Anaerolineales* is present in the GTDB-Tk classification tree these assignments are considered underclassified relative to the GTDB classification.


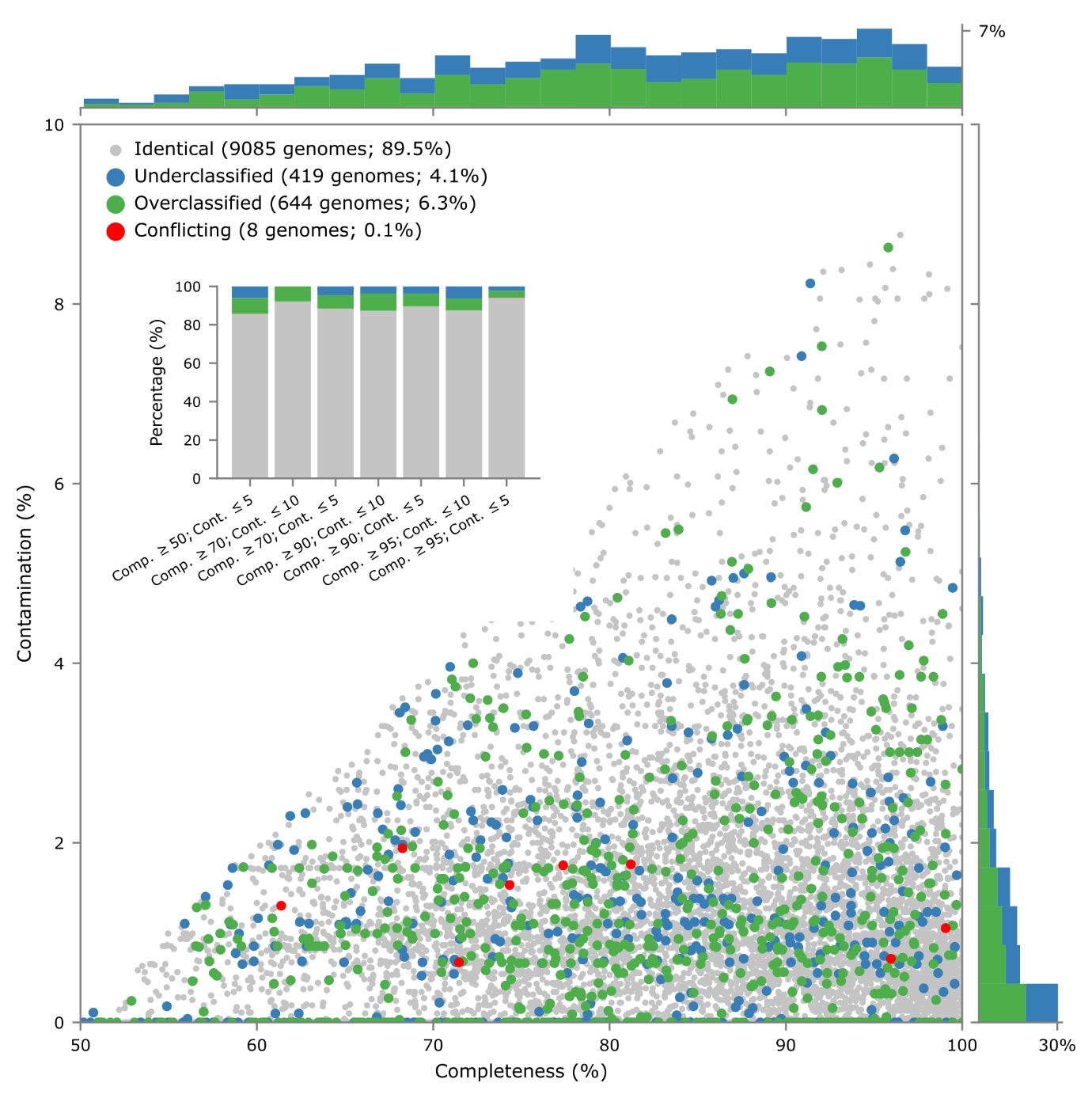


**Supplemental Figure S5**. Classification results for the 10,156 UBA MAGs as a function of their estimated completeness and contamination. Each point in the scatterplot represents a MAG which is colored to indicate the classification results of GTDB-Tk relative to GTDB. The histograms on the side of the scatterplot show the proportion of MAGs that were over- or under-classified for different completeness and contamination ranges. The inset plot indicates the percentage of identical, conflicting, overclassified, and underclassified MAGs for varying completeness and contamination criteria. Each MAG is assigned to the most restrictive criteria it satisfies (e.g., a 91% complete MAG with 4% contamination is only counted in the completeness ≥90% and contamination ≤5% result).


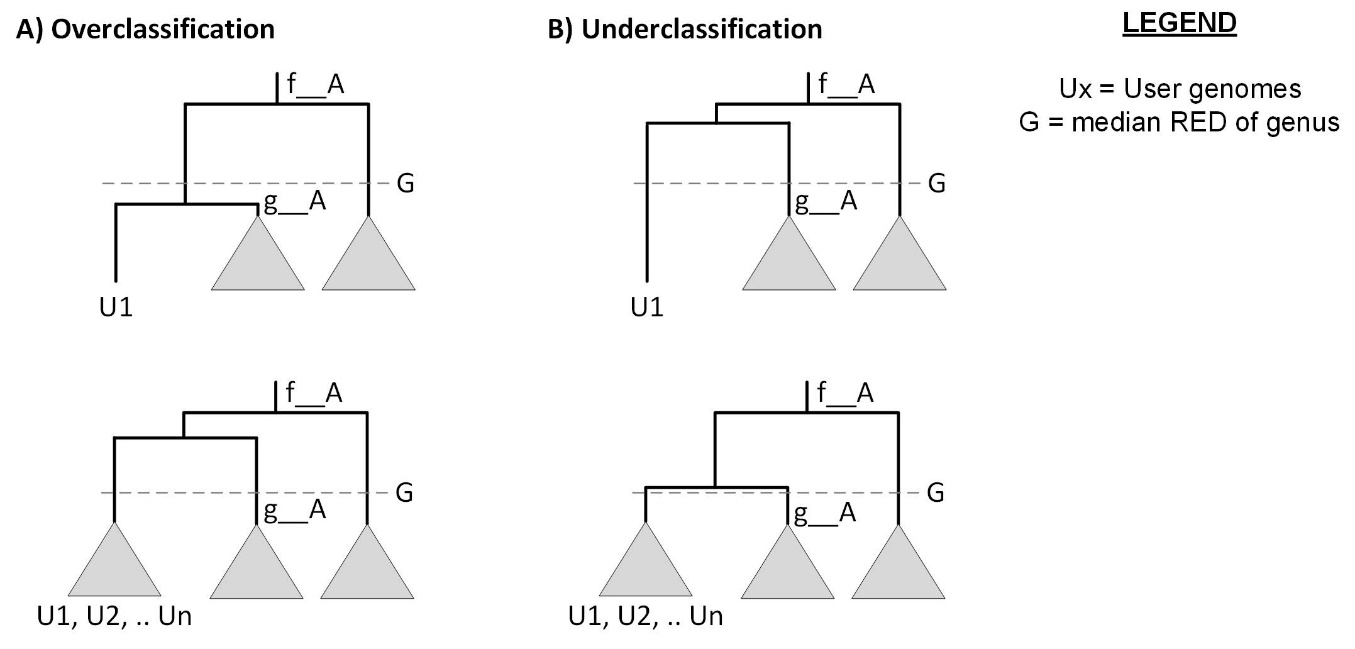


**Supplemental Figure S6**. Illustrative examples resulting in over- or under-classified genomes as a result of taxon sampling. (A) When the user genome U1 is considered in isolation it appears to be the most basal member of g__A. This is the assignment given by GTDB-Tk since it brings the RED value of g__A closer to the median genus-level RED value. After the addition of several new genomes a different taxonomic classification may be more suitable. Here, the addition of several genomes affiliated with U1 cause the branching point to move closer to f__A which can result in two distinct genera being defined in the GTDB. (B) When the user genome U1 is considered in isolation it appears to represent a new genus in the family f__A. This is the assignment given by GTDB-Tk since defining U1 to be in g__A would result in a RED value that deviates more substantially from the median genus-level RED value. After the addition of several user genomes affiliated with U1 the branching point may move closer to g__A resulting in these two lineages being defined as a single genus in the GTDB.


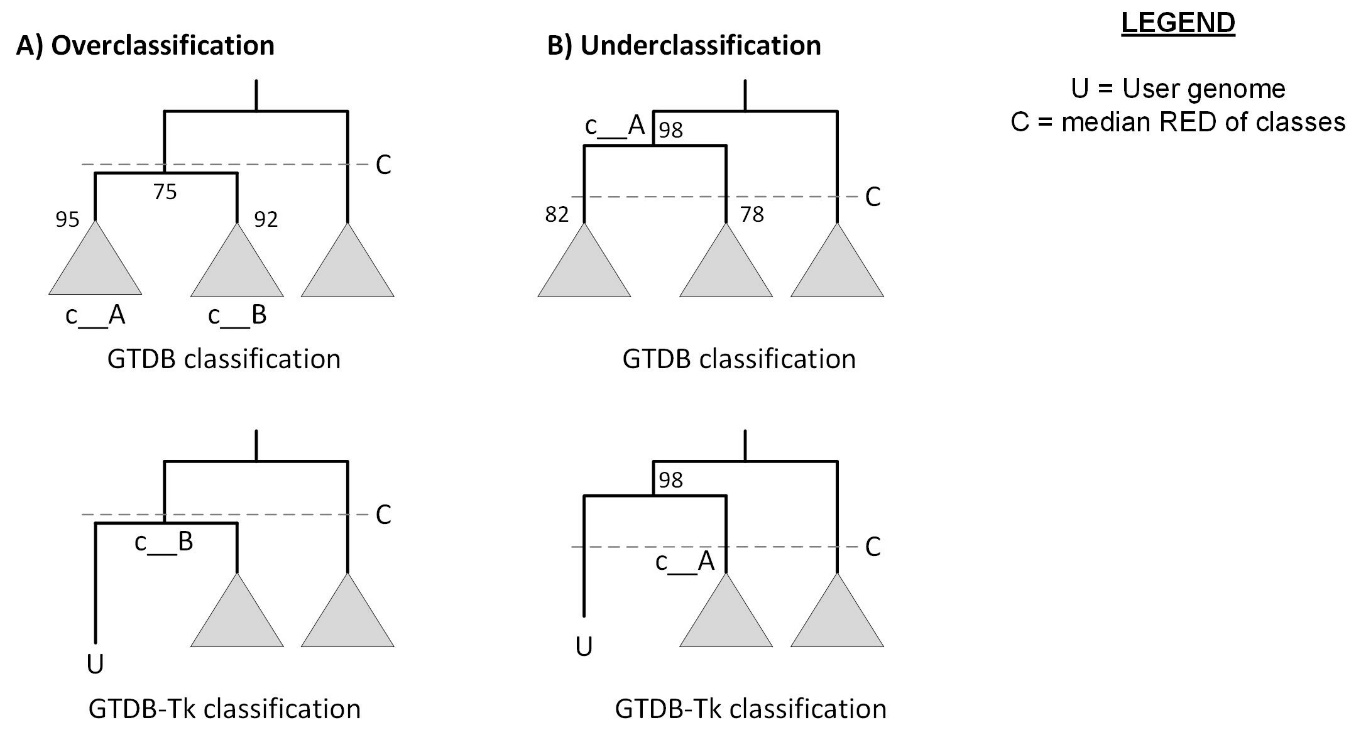


**Supplemental Figure S7**. Illustrative examples of over- and under-classified genomes as a result of differences between the rules used by GTDB-Tk and the manually curated GTDB. Numbers on internal nodes indicate bootstrap support values. (A) GTDB defines the two classes c__A and c__B at stable internal nodes instead of defining a single class at the internal node with only 75% support despite it being closer to the median class-level RED value. In the bottom tree, c__A has been pruned from the reference tree and a test user genome from c__A classified with GTDB-Tk. GTDB-Tk will classify this genome as the most basal member of c__B as this results in c__B being defined at a node closer to the median class-level RED value. (B) GTDB defines a single class at a stable internal node instead of defining two classes at less stable nodes. In the bottom tree, the left child lineage had been pruned from the reference tree and a test user genome from this lineage classified with GTDB-Tk. GTDB-Tk will classify this genome as a novel class since classifying it as the most basal genome within c__A would result in c__A being further away from the median class-level RED value.

**Supplemental Tables**

**Supplemental Table S1**. Classification performance on the 9,386 bacterial UBA genomes indicating the lowest rank for which classifications consistent with GTDB assignments can be obtained by GTDB-Tk.

|  | *No. genomes* | *Identical* | *Conflicting* | *Overclassified* | *Underclassified* |
| --- | --- | --- | --- | --- | --- |
| Domain | 20 | 15.0% | 0% | 85.0% | 0% |
| Phylum | 52 | 71.2% | 0% | 26.9% | 1.92% |
| Class | 123 | 54.5% | 0% | 42.3% | 3.25% |
| Order | 393 | 56.5% | 0.51% | 34.9% | 8.14% |
| Family | 1,646 | 70.8% | 0.24% | 22.6% | 6.32% |
| Genus | 3,545 | 92.4% | 0.06% | 0.71% | 6.83% |
| Species | 3,607 | 99.9% | 0% | 0% | 0.11% |
| *Total* | 9,386 | 89.2% | 0.09% | 6.57% | 4.12% |

**Supplemental Table S2**. Classification performance on the 770 archaeal UBA genomes indicating the lowest rank for which classifications consistent with GTDB assignments can be obtained by GTDB-Tk.

|  | *No. genomes* | *Identical* | *Conflicting* | *Overclassified* | *Underclassified* |
| --- | --- | --- | --- | --- | --- |
| Domain | 0 | 0% | 0% | 0% | 0% |
| Phylum | 4 | 50.0% | 0% | 50.0% | 0% |
| Class | 7 | 85.7% | 0% | 14.3% | 0% |
| Order | 49 | 79.6% | 0% | 12.2% | 8.16% |
| Family | 142 | 82.4% | 0% | 9.86% | 7.75% |
| Genus | 382 | 94.5% | 0% | 1.05% | 4.45% |
| Species | 186 | 100.0% | 0% | 0% | 0% |
| *Total* | 770 | 92.3% | 0% | 3.51% | 4.16% |

**Supplemental Table S3**. Information on the 1,071 MAGs with conflicting (8 MAGs), overclassified (644 MAGs), or underclassified (419 MAGs) assignments when comparing GTDB-Tk automated and GTDB manually curated taxonomic classifications.

(see Excel file)

**Supplemental Table S4**. Classification of the 10,156 UBA genomes when sampled without replacement into subsets of size 100, replicated 50 times. The results demonstrate that despite the non-deterministic nature of pplacer, classification is congruent with running all 10,156 UBA genomes in a single batch. The *trial* indicates which replicate the data refer to, *gtdb_taxonomy* is the true GTDB taxonomy, *trial_gtdbtk_classification* is the resulting GTDB-Tk classification from that trial run, *past_gtdbtk_classification* is the GTDB-Tk classification from the full 10,156 genomes run in a single batch, similarly *trial_pplacer_taxonomy* and *past_pplacer_classification* refer to the trial and single batch results respectively.

(see Excel file)
